# Support not stigma: redefining perinatal mental health care

**DOI:** 10.1016/j.lanepe.2024.100930

**Published:** 2024-05-06

**Authors:** The Lancet Regional Health – Europe

Historically, maternal mental health during the perinatal period, which spans from conception to 1 year after birth, is a neglected issue. Up to 85% of women experience so-called baby blues, characterised by symptoms such as prolonged bouts of crying, sadness, and anxiety. Globally, about 10% of pregnant women and 13% of women who have just given birth experience mental disorders, such as depression, anxiety, and psychosis, with higher prevalence observed in low- and middle-income countries. Death by suicide, which is strongly associated with mental health conditions, is a leading cause of mortality during the perinatal period in high-income countries, accounting for 5–20% of maternal deaths. The numbers are likely to be higher in low- and middle-income countries, where death by suicide often goes unreported and is overshadowed by inadequate care for obstetric complications.

Perinatal mental health conditions can significantly impact maternal and child wellbeing, hindering bonding, caregiving, and healthy development, which can have long-term consequences on the child's physical, cognitive, and emotional growth, affecting families and future generations.

Due to societal misconceptions about motherhood, fear of judgment, societal pressure to conform, and cultural taboos around mental health, perinatal mental health conditions are often stigmatised. This stigma can lead to feelings of shame and reluctance to seek help due to the fear of potential consequences, such as being viewed as unfit parents or encountering challenges in child custody disputes.

Urgent action is needed to address the stigma and systemic barriers surrounding maternal mental health, ensuring that all women receive comprehensive support and care during this important period of their lives.

For addressing stigma surrounding perinatal mental health conditions, essential approaches should prioritize raising awareness, educating about these conditions, fostering open discussions, and ensuring readily available support and resources for affected individuals.

Addressing systemic barriers to support mothers requires a comprehensive approach encompassing early identification of mental health conditions, preventive interventions, and effective treatments to promote maternal and child wellbeing throughout the perinatal period.

Screening for mental health symptoms is crucial to identify women who require assistance, since 70% of women hide or downplay their symptoms due to stigma and lack of awareness of their conditions, leading to unaccounted, undetected and untreated cases. National discrepancies in screening recommendations, assessment scales, and referral pathways hinder consensus on effective screening plans, impeding scalable actions for early detection and treatment. In the UK, approximately half of women with perinatal anxiety and depression remain undiagnosed, despite the existence of national guidelines recommending screening since 2014. Data show that one in six National Health Service trusts in England are not reporting whether the recommended mental health checks are being conducted. A proactive approach to identifying perinatal mental health symptoms is crucial for combating stigma, raising awareness, and overcoming this systemic barrier.

Preventive interventions, such as psychosocial and psychological therapies targeting perinatal mental health conditions are currently limited with regard to scalability and efficacy. A recent randomised, controlled phase 3 trial in a low-resource setting demonstrated promising results: risk of depression among pregnant women with mild anxiety who received cognitive behavioural therapy from non-specialists was reduced by 81% when compared with women who received enhanced care alone. Such effective preventive interventions, in low-resource settings, are crucial for addressing the unmet needs of vulnerable populations such as ethnic minorities, migrants, and those in war zones, who are at a significantly higher risk of perinatal mental health conditions, but are often unable to access adequate care.

Few approved pharmacological treatments exist for perinatal mental disorders, and conducting clinical trials for novel drugs in this population is challenging due to safety and ethical concerns for pregnant or breastfeeding women, hampering progress in research. However, in August, 2023, the US Food and Drug Administration approved the use of zuranolone, the first oral synthetic neurosteroid antidepressant that acts on GABA-A receptors, for postpartum depression. In contrast to traditional antidepressants, zuranolone is fast acting and is taken once daily for just 14 days, with optimal efficacy observed as early as 3 days. Before the approval of zuranolone, the only approved pharmacotherapy available for postpartum depression in the US was brexanolone, which requires a 60-h intravenous infusion but is limited to clinical trial use in Europe. Future research on neurosteroid antidepressants will be essential to address questions about long-term effects, potential side-effects on mothers and infants, and their superiority over routine treatments.

Furthermore, emerging complementary therapies such as light therapy, massage, and acupuncture, while promising for certain women, often require additional out-of-pocket payments due to limited coverage under statutory insurance. Further research is needed to explore their efficacy and cost-effectiveness to facilitate their integration into full statutory insurance coverage.

To shift from stigmatising perinatal mental health conditions to supporting mothers will require multidisciplinary approaches for early identification and prompt intervention. Collaborative action is imperative, requiring the commitment of clinicians, health-care providers, policy makers, and communities to prioritise maternal mental health within reproductive health care.

